# Supplementary material for: A universal metabolite repair enzyme removes a strong inhibitor of the TCA cycle
Source: Nat Commun. 2024 Jan 29;15:846. doi: 10.1038/s41467-024-45134-0 (PMC10825186; doi:10.1038/s41467-024-45134-0)
Supplement: Supplementary file 1 — Supplementary Information [file 41467_2024_45134_MOESM1_ESM.pdf]

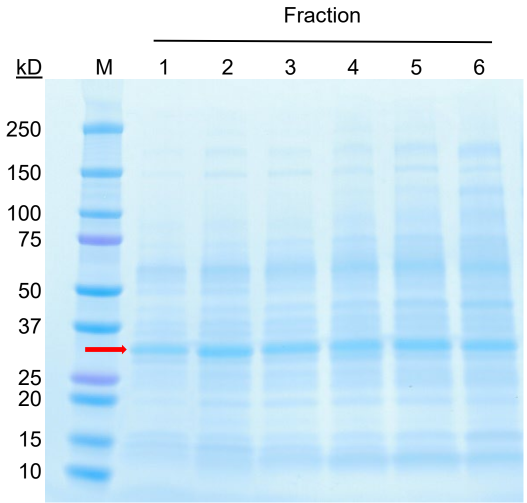

Fraction	1	2	3	4	5	6
total protein per assay, µg	0.12	0.51	0.44	0.41	0.4	0.65
specific activity, µmol min^{-1} mg^{-1}	25.4	21.1	20.7	20.2	18	9.4
expected purity	51%	42%	41%	40%	36%	19%

Fig. S1. Partial purification of OAT1 from bovine heart mitochondrial matrix proteins.

The six 5.0 mL fractions eluted from the hydroxyapatite column with 10 mM potassium phosphate were analyzed by SDS-PAGE and direct enol→keto OAT enzyme assays. For SDS-PAGE, samples were resolved on Bio-Rad Mini-Protean TGX gels and stained with Bio-Rad Bio-Safe Coomassie G250. The major band at ~32 kD is indicated with a red arrow. Enzyme assays were performed with 5 µL of each fraction. The protein content of each fraction was determined by Bradford dye-binding assays. Expected purity was based on the previously reported specific activity for purified OAT1 of 50 µmol min^{-1} mg^{-1}.

>Bovine_FAHD2A (UniProt ID: F1MLX0)
MLGSSGRRLLTTVLQAQRWPFQPSRNMR**LVQFQAPHLAGPHLGLESGNGGGVIDLNAFEP**
TLPKTMVEFLEQGEATLSVVRRALATQLPVLPRSEVTFLAPVTRPDKVVCVGMNYADHCR
EQNVPVPKEPIIFSKFASAIVGPYDNIILPPESQEVDWEVELAVVIGKRGK**YIKATDAMA**
HVAGFTVAHDVSARDWQMGRNGKQWLLGKTFDTFCPLGPALVTKDSVADPHNLKICCRVN
GEVMQSSNTNQMVFKTEELITWVSQFVTLYPGDIILTGTPPGVGVFRKPPVFLKKGDEVQ
CEIEELGVIINKVV

>Bovine_FAHD2B (UniProt ID: Q2KIB0)
MLGSSGRRLLTTVLQAQRWPFQPSRDMR**LVQFQAPHLAGPHLGLESGNGGGVIDLNAFEP**
TLPKTMVEFLEQGEATLSVVRRALATQLPVLPRSEVTFLAPVTRPDKVVCVGMNYADHCR
EQNVPVPKEPIIFSKFASAIVGPYDNIILPPESQEVDWEVELAVVIGKRGK**YIKATDAMA**
HVAGFTVAHDVSARDWQMGRNGKQWLLGKTFDTFCPLGPALVTKDSVADPHNLKICCRVN
GELMQSSNTNQMVFKTEELITWVSQFVTLYPGDIILTGTPPGVGVFRKPPVFLKKGDEVQ
CEIEELGVIINKVV

LVQFQAPHLAGPHLGLESGNGGGVIDLNAFEPTLPK
TMVEFLEQGEATLSVVR
TMVEFLEQGEATLSVVRR
RAALATQLPVLPR
AALATQLPVLPR
AALATQLPVLPRSEVTFLAPVTRPDK
SEVTFLAPVTRPDK
SEVTFLAPVTRPDKVVCVGMNYADHCR
VVCVGMNYADHCR
VVCVGMNYADHCREQNVPVPK
EQNVPVPK
EQNVPVPKEPIIFSK
EPIIFSK
FASAIVGPYDNIILPPESQEVDWEVELAVVIGK
FASAIVGPYDNIILPPESQEVDWEVELAVVIGKR
YIKATDAMAHVAGFTVAHDVSAR
ATDAMAHVAGFTVAHDVSAR
ATDAMAHVAGFTVAHDVSARDWQMGR
DWQMGR
DWQMGRNGK
NGKQWLLGK
TFDTFCPLGPALVTK
TFDTFCPLGPALVTKDSVADPHNLK
DSVADPHNLK
DSVADPHNLKICCR
ICCRVNGEVMQSSNTNQMVFK
VNGELMQSSNTNQMVFK
VNGEVMQSSNTNQMVFK
TEELITWVSQFVTLYPGDIILTGTPPGVGVFR
KPPVFLK
KPPVFLKK
KGDEVQCEIEELGVIINK
GDEVQCEIEELGVIINK

Fig. S2. Summary of MS proteomic sequencing results from trypsin-digested bovine OAT1
The ~32-kD protein judged to be bovine OAT1 was excised from an SDS-PAGE gel and submitted for trypsin digest and MS analysis by the University of Minnesota Center for Mass Spectrometry & Proteomics and the data was processed with Sequest set up to search uniprot_bos_taurus9913_UP0000091320200622_unipr_cRAP.fasta. Of the total spectra detected, >68% matched to bovine FAHD2A and/or FAHD2B, which differ only by a single amino acid (V or L) at position 243. 33 unique peptides matching these proteins were detected. No other protein matched to more than 4% of detected spectra. The amino acid sequences of FAHD2A and FAHD2B are shown with regions containing detected peptides in bold font and the single amino acid difference at position 243 colored red. Unique detected peptides are listed. The N-terminal 29 amino acids for which no peptides were detected closely corresponds to the predicted mitochondrial targeting peptide. Two unique peptides showing V243 (underlined) and one unique peptide showing L243 (underlined) were detected, indicating that both Bovine FAHD2A and FAHD2B were present in the sample.

```
           1                                                                                                                                      130
  Bs YisK        MKFA TGELYNRMFV GLIIDDEKIM DLQKAEKKLF ELETIPGSLI ECIAEGDKFV AHARQLAEWA KKPNDELGSF MYSL------ ---SEVKLHA PIPKPSKNII CIGKNYRDHA IEMGSEADIP
Bt FAHD2A  MLGSSGRRLL TTVLQAQRWP FQPSRDMRLV QFQAPHLAGP HLGLESGNGG GVIDLNAFEP TLPKTMVEFL EQGEATLSVV RRALATQLPV LPRSEVTFLA PVTRPDK-VV CVGMNYADHC REQN--VPVP
Hs FAHD2A  MLVSGRRRLL TVLLQAQKWP FQPSRDMRLV QFRAPHLVGP HLGLETGNGG GVINLNAFDP TLPKTMTQFL EQGEATLSVA RRALAAQLPV LPRSEVTFLA PVTRPDK-VV CVGMNYVDHC KEQN--VPVP
Hs FAHD2B  MLVSGRRRLL TALLQAQKWP FQPSRDMRLV QFRAPHLVGP HLGLETGNGG GVINLNAFDP TLPKTMTQFL EQGEATLSVA RRALAAQLPV LPWSEVTFLA PVTWPDK-VV CVGMNYVDHC KEQN--VPVP
  Mm FAHD                                                                                                                MEIKPSK-IV CAGLNYKDHA EELK--MKLP
 Sc FMP41                                                                                                                       MSY NYLKAARKVI CIGRNYAAHI KELN--NSTP
  Ec YcgM                                                                                                            MYQHHNWQGA LLDYPVSKVV CVGSNYAKHI KEMG--SAVP
 Hs FAHD1                                                                                                          M GIMAASRPLS RFWEWGKNIV CVGRNYADHV REMR--SAVL
At FAHD1A                                                                                                               MATSMIQ RMFKQGTKIV CVGRNYAAHA KELG--NAVP
At FAHD1B                                                                                                               MATSMIQ RLFKQGTKIV GVGLNYASHA KELG--NALP

           131                                                                                                 #   #                                  260
  Bs YisK  EHPMVFTKSP VTVTG----- ---------- ---------- HGDIVKSHEE VTSQLDYEGE LAVVIGKSGT RISK---EDA YDHVFGYTIV NDITARDLQ- -KRH--KQFF IGKSLDTTCP MGPVLVHKSS
Bt FAHD2A  KEPIIFSKFA SAIVG----- ---------- ---------- PYDNIILPPE -SQKVDWEVE LAVVIGKRGK YIKA---TDA MAHVAGFTVA HDVSARDWQ- -MGRNGKQWL LGKTFDTFCP LGPALVTKDS
Hs FAHD2A  KEPIIFSKFA SSIVG----- ---------- ---------- PYDEVVLPPQ -SQEVDWEVE LAVVIGKKGK HIKA---TDA MAHVAGFTVA HDVSARDWQ- -MRRNGKQWL LGKTFDTFCP LGPALVTKDS
Hs FAHD2B  KEPIIFSKFA SSIVG----- ---------- ---------- PYDEVVLPPQ -SQEVDWEVE LAVVIGKKGK HIKA---TDA MAHVAGFTVA HDVSARDWL- -TRRNGKQWL LGKTFDTFCP LGPALVTKDS
  Mm FAHD  KEPVIFLKPT SSIIY----- ---------- ---------- NEESIKIPKA -SERVDYEAE LAIVIGKTCK SVKK---EDA KNYIKGFTIL NDVTARDLQ- -EKD--GQWT RAKSFDTFCP VGPKIV---S
 Sc FMP41  KQPFFFLKPT SSIVTPLSSS LVKTTRPANS TFNGLNEDGT NPGPIFIPRG -V-KVHHEIE LALIVSKHLS NVTKMKPEEV YDSISGVALA LDLTARNVQD EAKKKGLPWT ISKGFDTFMP ISAIVSREKF
  Ec YcgM  EEPVLFIKPE TALCD----- ---------- ---------- LRQPLAIPSD -FGSVHHEVE LAVLIGATLR QATE---EHV RKAIAGYGVA LDLTLRDVQG KMKKAGQPWE KAKAFDNSCP LSGFIPAAEF
 Hs FAHD1  SEPVLFLKPS TAYAP----- ---------- ---------- EGSPILMPAY -TRNLHHELE LGVVMGKRCR AVPE---AAA MDYVGGYALC LDMTARDVQD ECKKKGLPWT LAKSFTASCP VSAFVPKEKI
At FAHD1A  KEPVIFLKPT SSYLE----- ---------- ---------- NGGTIEIPHP -LDSLHHEVE LALVIGQKAR DVPE---SIA MDYIGGYAVA LDMTARELQA SAKASGLPWT VAKGQDTFTP ISSVLPKAMV
At FAHD1B  KDPIVFLKPT SSYLE----- ---------- ---------- NGGTIEIPHP -LDSLHHEVE LAVVIGQKAR DVPE---RLA MNYIGGYALA LDMTARELQV SAMASGLPCT LAKGQDTFTP ISSVLPKAMV

           261                                                                                                        360
  Bs YisK  IQEPERLK-- --VETRVNGE LRQSGSASDM IFSIPELIET LSKGMTLEAG DIIATGTPSG VGKGFTPPKF LRSGDKIDIT IDPIGTLSNQ IG
Bt FAHD2A  VADPHNLK-- --ICCRVNGE VMQSSNTNQM VFKTEELITW VSQFVTLYPG DIILTGTPPG VGVFRKPPVF LKKGDEVQCE IEELGVIINK VV
Hs FAHD2A  VADPHNLK-- --ICCRVNGE VVQSGNTNQM VFKTEDLIAW VSQFVTFYPG DVILTGTPPG VGVFRKPPVF LKKGDEVQCE IEELGVIINK VV
Hs FAHD2B  VADPHNLK-- --ICCRVNGE VVQSSNTNQM VFKTEDLIAW VSQFVTFYPG DVILTGTPPG VGVFRKPPVF LKKGDEVQCE IEELGVIINK VV
  Mm FAHD  GIDANNLK-- --IQLRVNGK IKQDSNTNNM IFSVEELVEF VSSIMTLYPD DIISTGTPPG IGK------- LEKGDIVEIE IENIGILKNF VE
 Sc FMP41  SSYKSNLQDI FRVKCSVNGQ LRQDGGTNLM LHPLHKILQH ISTMISLEPG DIILTGTPAG VGELKPGDHV HCELLQNNDN IVDMNFECEN RPGPYEFRET
  Ec YcgM  TGDPQNTT-- --LSLSVNGE QRQQGTTADM IHKIVPLIAY MSKFFTLKAG DVVLTGTPDG VGPLQSGD-- --ELTVTFDG HSLTTRVL
 Hs FAHD1  P-DPHKLK-- --LWLKVNGE LRQEGETSSM IFSIPYIISY VSKIITLEEG DIILTGTPKG VGPVKEND-- --EIEAGIHG LVSMTFKVEK PEY
At FAHD1A  R-DPDNLE-- --LWLKVDGE TRQKGLTKDM IFKVPYLISY ISSIMTLY-G DVILTGTPEG VGPVKIGQ-- --KITAGITG LSEVQFDVER RVKPLS
At FAHD1B  L-DPNNLE-- --LWLKVDDE TRQKGWTKDM IFKVPYLISY ISSVMTLFKG DVILTGTPEG IGPVKIGQ-- --KITAGITG LSEVQFDVGR RLKPLLR
```

Fig. S3. Amino acid alignment of FAHD proteins used in this study.

Amino acid sequences of FAHD proteins used in this study were aligned with MultAlin software ("Multiple sequence alignment with hierarchical clustering" F. CORPET, 1988, Nucl. Acids Res., 16 (22), 10881-10890). Conserved residues are colored red and residues conserved in at least 50% of sequences are colored blue. Residues mutated in *H. sapiens* FAHD1 to create a catalytically inactive M2-FAHD1 enzyme are highlighted yellow and indicated above the alignment (#).

A

```
 1                                                          50
FAHD1   MGIMAASRPL SRFWEWGKNI VCVGRNYADH VREMRSAVLS EPVLFLKPST AYAPEGSPIL MPAYTRNLHH ELELGVVMGK RCRAVPEAAA MDYVGGYALC
   M2   MGIMAASRPL SRFWEWGKNI VCVGRNYADH VREMRSAVLS EPVLFLKPST AYAPEGSPIL MPAYTRNLHH ELELGVVMGK RCRAVPEAAA MDYVGGYALC

        101                                                    150
FAHD1   LDMTARDVQD ECKKKGLPWT LAKSFTASCP VSAFVPKEKI PDPHKLKLWL KVNGELRQEG ETSSMIFSIP YIISYVSKII TLEEGDIILT GTPKGVGPVK
   M2   LAMTAADVQD ECKKKGLPWT LAKSFTASCP VSAFVPKEKI PDPHKLKLWL KVNGELRQEG ETSSMIFSIP YIISYVSKII TLEEGDIILT GTPKGVGPVK

        201                  224
FAHD1   ENDEIEAGIH GLVSMTFKVE KPEY
   M2   ENDEIEAGIH GLVSMTFKVE KPEY
```

B

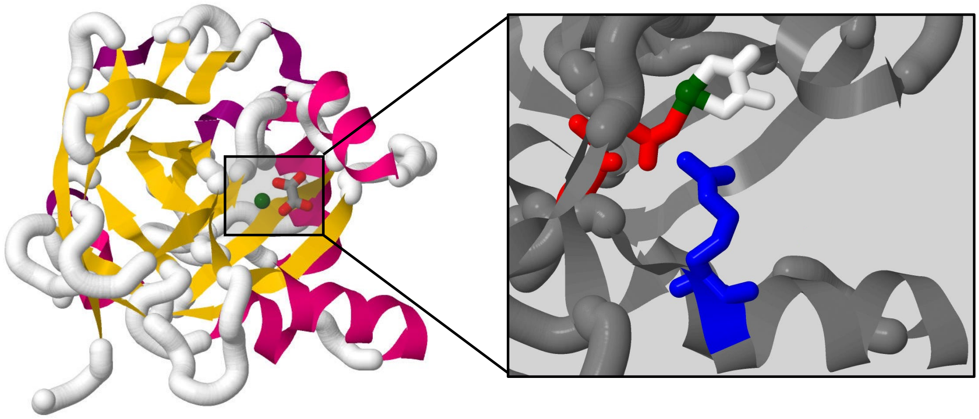

C

Enzyme	Activity (μmol·min^{-1}·mg^{-1})
native FAHD1	69.0 ± 10.0
M2-FADH1	0.022 ± 0.013

Fig. S4. Human FAHD1 with D102A and R106A mutations has very minor OAT activity. (**A**) Amino acid alignment of native human FAHD1 and M2-FADH1 with mutated amino acids indicated with yellow highlighting and colored red. (**B**) Crystal structure of human FAHD1 bound to oxalate, accessed from PDB entry 6FOG. Subunit A of FAHD1 is shown with the active site enlarged for clarity. Asp102 (red) appears to bind a divalent cation (green) that facilitates oxalate (white) binding. Arg106 (blue) also appears to be involved in oxalate binding. (**C**) OAT activity of M2-FAHD1 was determined with the MDH coupled assay described in the methods, except that the amount of M2-FADH enzyme was increased to 5 μg. Our results indicate that the D102A and R106A mutations decrease OAT activity by >99%.

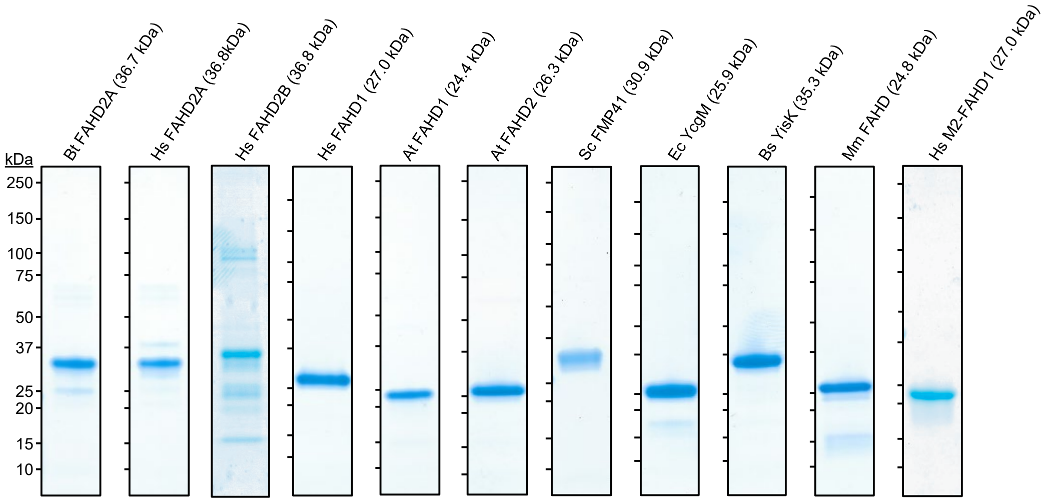

Fig. S5. Recombinant FAHD enzymes were purified to near homogeneity

N-terminally hexahistidine-tagged proteins were expressed in *E. coli* and purified by affinity chromatography. Total protein (2 µg) from each preparation was analyzed by SDS-PAGE with Coomassie staining. The migration position of molecular weight standards is indicated with tick marks. Each protein preparation was judged to be ≥95% pure.

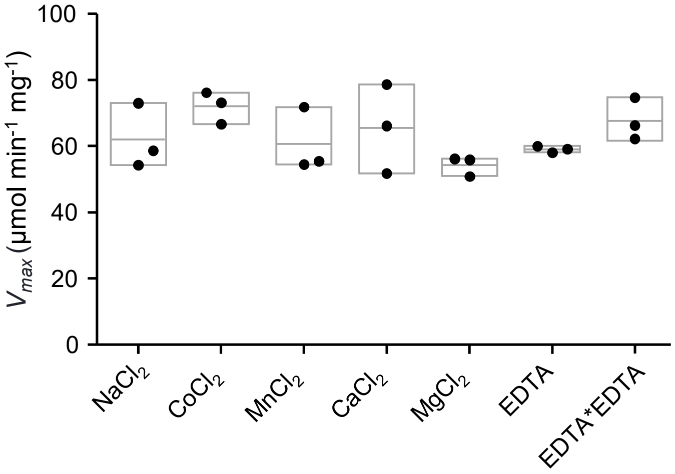

Fig. S6. The effect of divalent cations on OAT enzyme activity of human FAHD1

The MDH coupled assay was used measure OAT activity in the enol→keto direction with the addition of 1 mM divalent cation chloride salt or EDTA or 1.5 mM sodium chloride. EDTA*EDTA indicates enzyme was incubated with 5 mM EDTA on ice for 30 minutes prior to analysis, then assayed with 1 mM EDTA. Enzyme velocities at various substrate concentrations were determined and V_{max} was calculated by fitting data to the Michaelis–Menten equation using GraphPad Prism Software. One-way analysis of variance showed no significant difference between samples.

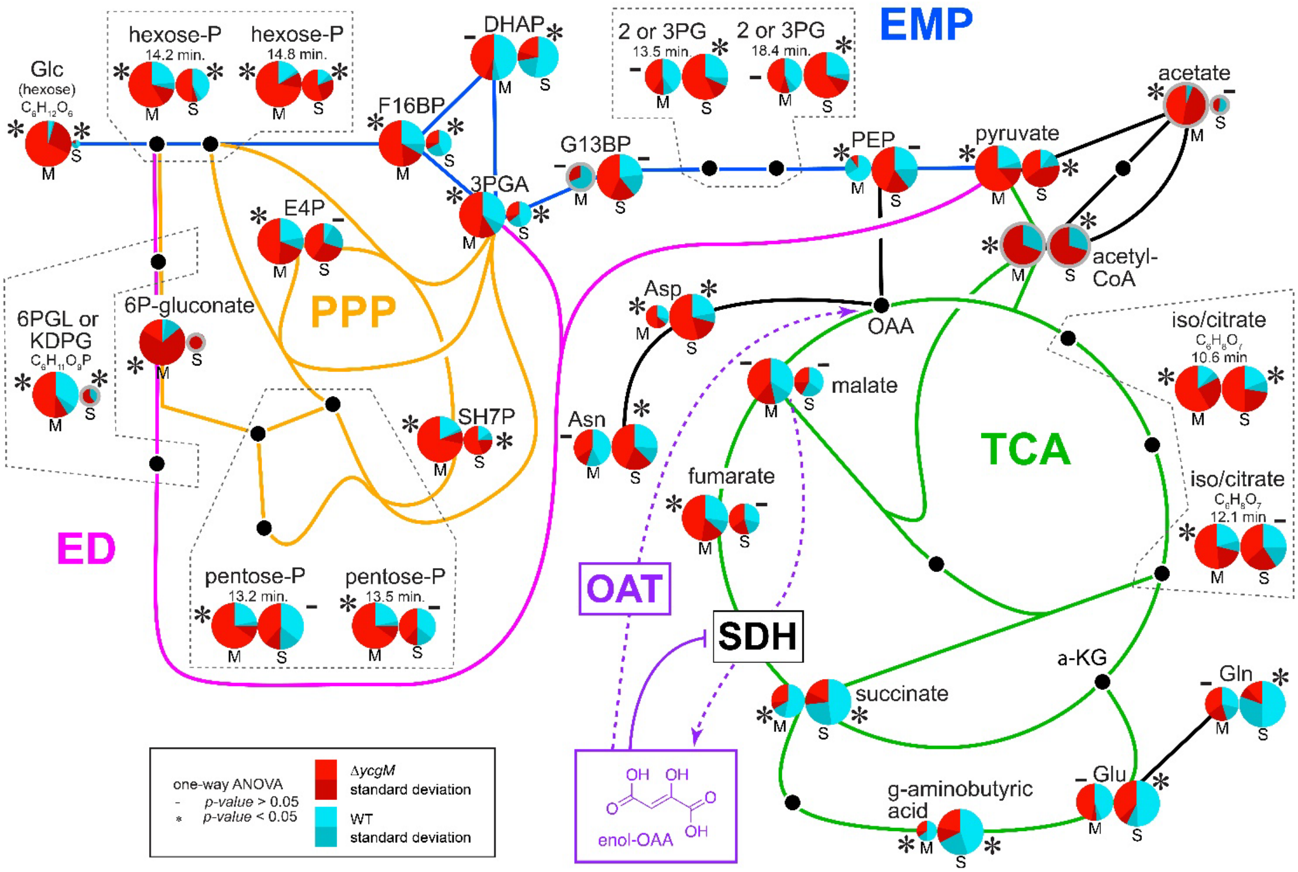

Fig. S7. Comparison of mid-log and early stationary growth phase metabolite profiles

The relative abundance of respiratory intermediates at mid-log (M) and early-stationary (S) growth phases in *ΔycgM* (red slices) or wild type (blue slices) *E. coli* determined by isotope ratio HILIC-MS analysis. Pie charts outlined in gray show relative abundance determined by comparing peak heights. Separate M & S pie charts have been scaled such that the change in chart area represents the relative change in the metabolite abundance between the two growth phases (calculations are given in table S1 tab 3 columns I-M). Data represents mean and SD, n=4x2, with statistical significance indicated (-, p>0.05; *. p<0.05; one-way ANOVA; values are given in table S1 tab 4

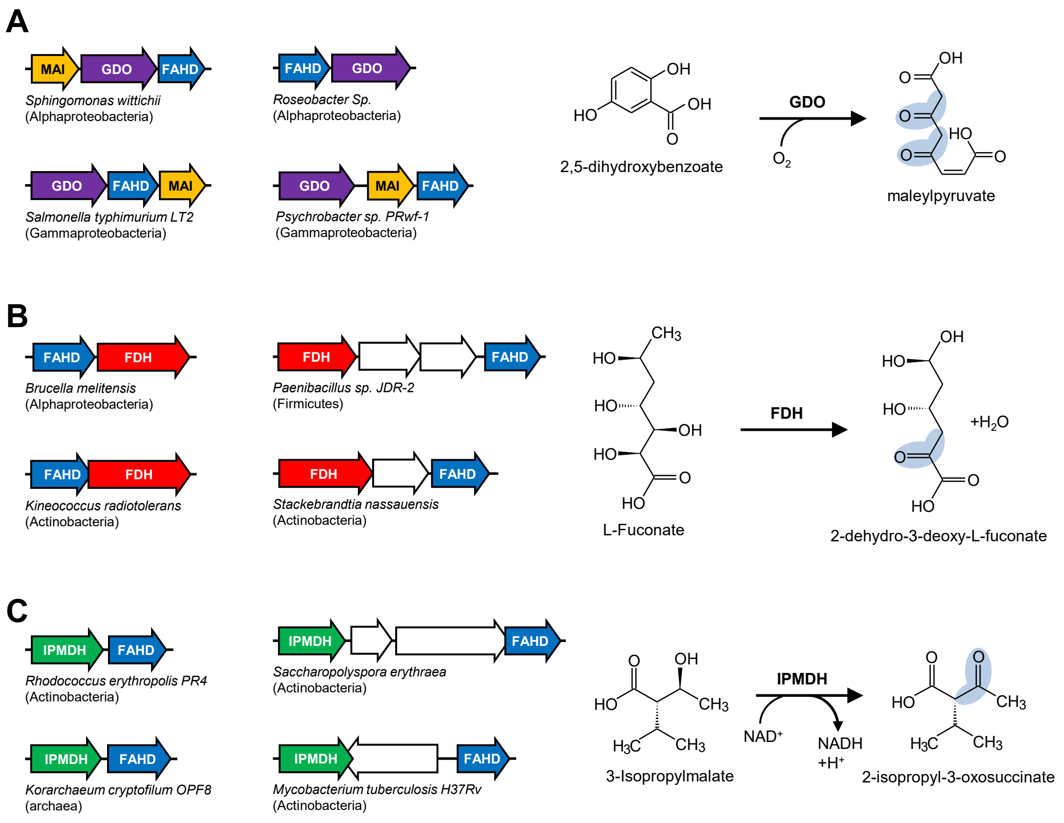

Fig. S8. FAHD genes associate with genes encoding enzymes that act on enol-keto-tautomerizable metabolites

(**A**) FAHD genes cluster on the chromosome with genes encoding gentisate 1,2-dioxygenase (GDO) (EC 1.13.11.4) in diverse microbes and in multiple orientations, indicating a functional association. The reaction catalyzed by GDO is shown with enol-keto tautomerizable functional groups shaded blue.
(**B**) FAHD genes cluster on the chromosome with genes encoding L-fuconate dehydratase (FDH) (EC 4.2.1.68) in diverse microbes and in multiple orientations, indicating a functional association. The reaction catalyzed by FDH is shown with the enol-keto tautomerizable functional group shaded blue.
(**C**) FAHD genes cluster on the chromosome with genes encoding 3-isopropylmalate dehydrogenase (IPMDH) (EC 1.1.1.85) in diverse microbes and in multiple orientations, indicating a functional association. The reaction catalyzed by IPMDH is shown with the enol-keto tautomerizable functional group shaded blue.

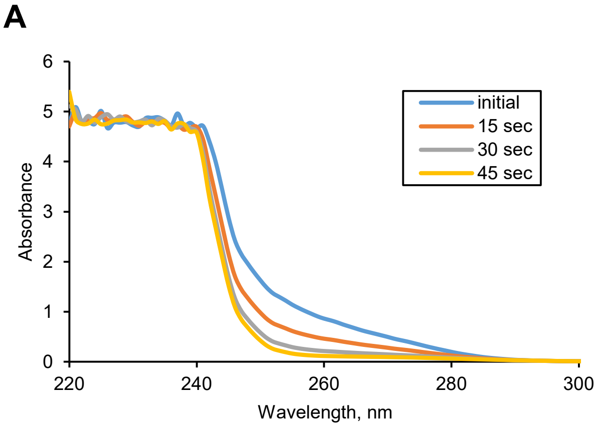

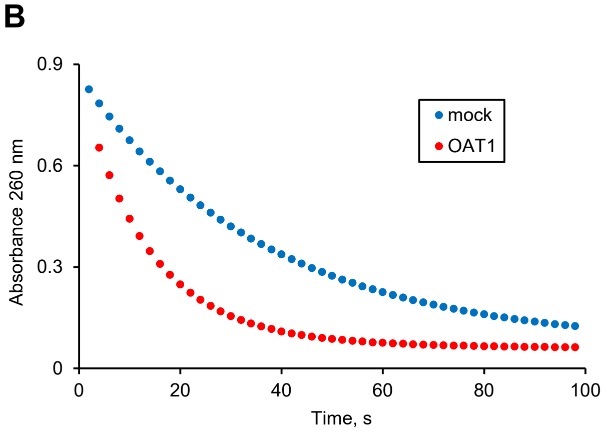

Fig. S9. FAHD enzymes have acetoacetate enol-keto tautomerase activity

(**A**) The spontaneous enol-keto tautomerization of acetoacetate can be detected spectrophotometrically. Acetoacetate was dissolved in dimethylformamide to 20 mM and 2 µL was added to 98 µL of 2 mM Tris-HCl, pH 9.0 while monitoring absorbance between 220 and 300 nm. Wavelength scans were recorded immediately after addition of acetoacetate and after 15, 30 and 45 s.

(**B**) OAT1 enzymes increase the rate of acetoacetate enol-keto tautomerization. Assays (100 µL total) contained 2 mM Tris-HCl, pH 9.0 with or without 5 µg of *Methanococcus* FAHD enzyme and were started by adding 2 µL of 20 mM acetoacetate in DMF. Absorbance at 260 nm was recorded every 2 s.

>*H. sapiens* FAHD2A_optimized

ATGCTGGTGTCTGGTAGAAGAAGGTTACTCACAGTTCTGCTGCAGGCTCAGAAGTGGCCCTTTCAACCCTCCAGAGACATGAGACT
AGTGCAGTTCCGGGCACCCCACCTGGTGGGGCCTCACTTGGGCCTGGAGACAGGGAATGGTGGAGGGGTTATCAACCTCAATGCCT
TTGACCCCACACTCCCGAAGACGATGACGCAGTTCCTAGAGCAGGGAGAGGCCACCCTCTCAGTGGCAAGAAGAGCCCTGGCTGCC
CAGTTGCCAGTCCTACCACGGTCGGAGGTAACCTTCCTGGCTCCAGTCACACGACCAGATAAGGTGGTGTGTGTGGGCATGAATTA
TGTGGACCACTGCAAAGAACAGAACGTGCCCGTGCCCAAGGAGCCCATCATCTTCAGCAAGTTTGCCAGCTCCATCGTGGGGCCCT
ATGATGAGGTGGTCCTCCCACCACAGAGCCAGGAGGTAGATTGGGAAGTGGAGCTGGCCGTGGTCATTGGAAAGAAAGGCAAGCAC
ATCAAGGCCACAGATGCTATGGCCCACGTGGCCGGCTTCACTGTGGCTCATGACGTGAGTGCTCGTGACTGGCAAATGAGACGTAA
TGGGAAACAATGGCTGCTGGGAAAAACCTTCGACACCTTCTGCCCTCTGGGCCCTGCCTTGGTGACCAAGGACAGTGTAGCAGATC
CACACAACTTAAAGATCTGCTGCCGAGTGAATGGGGAAGTGGTCCAGAGCGGCAACACCAACCAGATGGTATTCAAGACAGAGGAC
CTGATAGCCTGGGTCTCCCAGTTTGTTACCTTTTACCCAGGGGATGTCATCCTAACTGGGAC**A**CC**A**CCAGGTGTCGGTGTATTCAG
GAAACCTCCTGTCTTTCTCAAGAAGGGGGATGAAGTCCAGTGTGAGATTGAAGAACTAGGTGTCATCATCAACAAGGTGGTGTGA

>*H. sapiens* FAHD2B_optimized

ATGTTAGTATCAGGACGAAGAAGGTTGCTAACTGCCTTACTGCAGGCACAGAAATGGCCTTTTCAGCCGAGCCGTGACATGCGTTT
GGTGCAATTCCGCGCGCCACACCTGGTTGGTCCGCACCTGGGCCTGGAGACAGGCAACGGTGGTGGGGTGATCAATCTAAACGCGT
TTGACCCGACCCTGCCCAAGACGATGACCCAATTCTTGGAACAGGGTGAGGCGACCCTGTCGGTCGCCCGCCGTGCTTTAGCGGCA
CAGTTGCCGGTGCTGCCGTGGTCTGAAGTTACGTTCTTGGCCCCAGTGACCTGGCCGGATAAAGTGGTTTGTGTTGGCATGAACTA
TGTTGACCACTGCAAAGAGCAAAACGTGCCGGTCCCGAAAGAACCGATTATCTTTAGCAAGTTCGCGAGCTCCATTGTTGGTCCGT
ACGACGAGGTCGTGCTGCCGCCACAGAGCCAAGAAGTAGATTGGGAAGTTGAGCTGGCGGTTGTGATCGGCAAGAAAGGCAAGCAC
ATTAAAGCGACCGATGCTATGGCACATGTTGCCGGTTTTACCGTAGCGCATGATGTAAGCGCAAGAGACTGGTTGACGCGTCGCAA
CGGCAAGCAGTGGTTGCTGGGTAAGACCTTTGACACCTTCTGCCCGCTGGGTCCGGCGCTCGTGACGAAAGACAGCGTTGCTGATC
CGCATAATCTGAAAATCTGCTGCCGTGTTAATGGCGAGGTCGTGCAATCTTCAAATACCAACCAGATGGTTTTCAAGACCGAAGAT
CTGATTGCTTGGGTTTCCCAGTTCGTGACCTTCTACCCGGGTGACGTGATCCTGACCGGTACTCCGCCGGGTGTGGGCGTGTTTCG
TAAACCGCCGGTCTTTCTTAAGAAGGGCGATGAGGTGCAATGTGAAATTGAAGAGCTGGGCGTTATCATCAACAAAGTGGTCTAA

>*H. sapiens* M2-FAHD1 (D102A R106A)

ATGGGAATCATGGCAGCATCCAGGCCATTGTCCCGCTTCTGGGAGTGGGGAAAGAACATCGTCTGCGTGGGGAGGAACTACGCGGA
CCACGTCAGGGAGATGCGCAGCGCGGTGTTGAGCGAGCCCGTGCTGTTCCTGAAGCCGTCCACGGCCTACGCGCCCGAGGGCTCGC
CCATCCTCATGCCCGCGTACACTCGCAACCTGCACCACGAGCTGGAGCTGGGCGTGGTGATGGGCAAGCGCTGCCGCGCAGTCCCC
GAGGCTGCGGCCATGGACTACGTGGGCGGCTATGCCCTGTGCCTGG**C**TATGACCGCC**GC**GGACGTGCAGGACGAGTGCAAGAAGAA
GGGGCTGCCCTGGACTCTGGCGAAGAGCTTCACGGCGTCCTGCCCGGTCAGCGCGTTCGTGCCCAAGGAGAAGATCCCTGACCCTC
ACAAGCTGAAGCTCTGGCTCAAGGTCAACGGCGAACTCAGACAGGAGGGTGAGACATCCTCCATGATTTTTTCCATCCCCTACATC
ATCAGCTATGTTTCTAAGATCATAACCTTGGAAGAAGGAGATATTATCTTGACTGGGACGCCAAAGGGAGTTGGACCGGTTAAAGA
AAACGATGAGATCGAGGCTGGCATACACGGGCTGGTCAGTATGACATTTAAAGTGGAAAAGCCAGAATATTGA

Fig. S10. Nucleotide sequences used to express human FAHD2A, FAHD2B, and M2-FAHD1
Nucleotide sequences were synthesized by Integrated DNA Technologies using gBlocks assembly. For FAHD2A, two cytosine residues were changed to adenines (bold and underlined) to remove a stretch of seven cytosine residues without changing the encoded amino acid sequence. For FAHD2B, the sequence was codon optimized to improve heterologous expression. For M2-FAHD1, three nucleotides were changed (bold and underlined) to introduce D102A and R106A mutations. Nucleotides encoding predicted mitochondrial targeting peptides are highlighted yellow and weren't included in expression constructs.

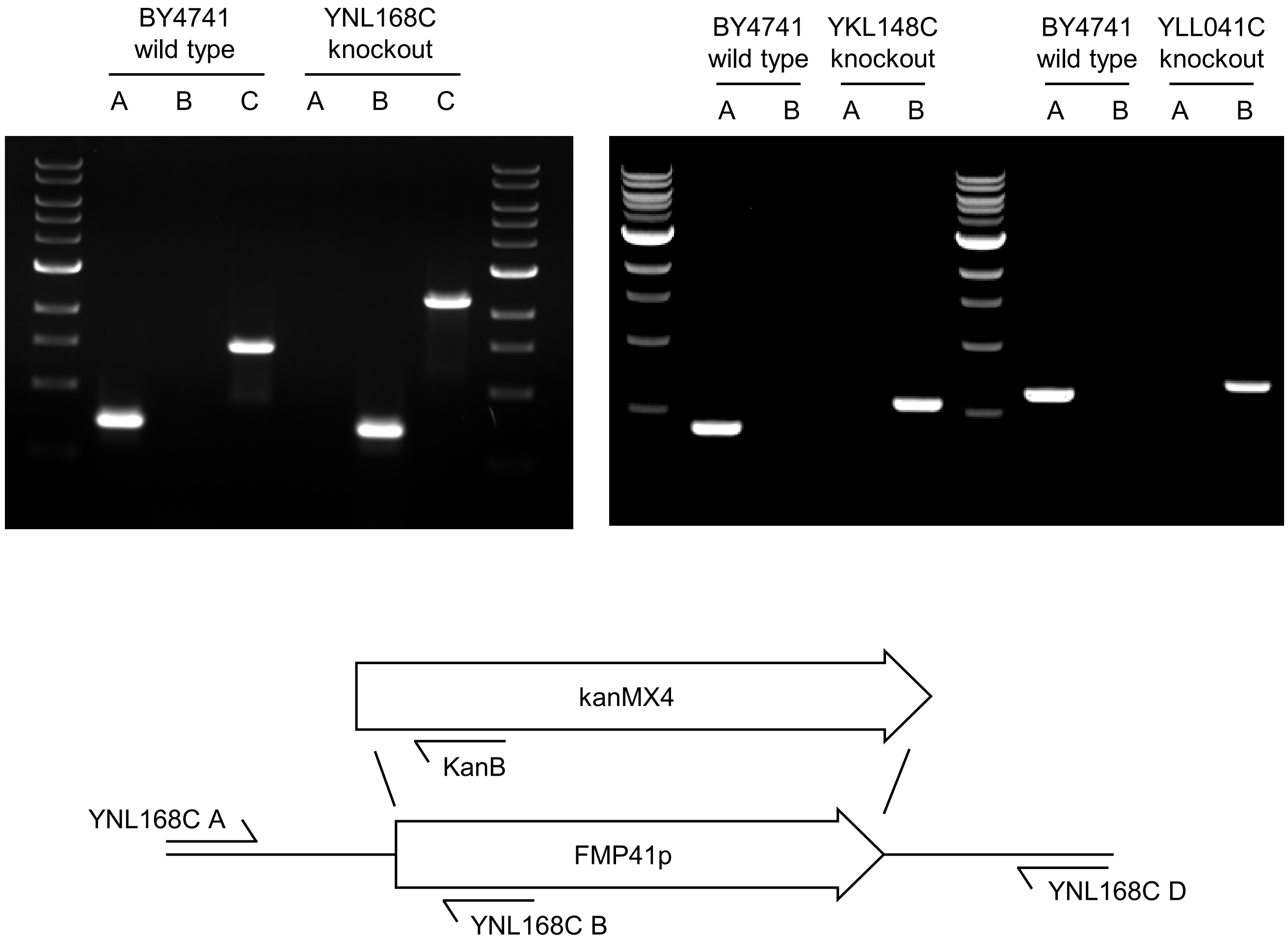

Fig. S11. Verification of *S. cerevisiae Δfmp41p* mutant this study. Genomic DNA was isolated from wild-type *S. cerevisiae* (BY4741) and knockout strains YNL168C (FAHD1), YKL148C (SDH1), and YLL041C (SDH2) and used in PCR reactions with either primer set A (YNL168C A and YNL168C B), B (YNL168C A and KanB) or C (YNL168C A and YNL168C D). Reaction products were analyzed by electrophoresis on a 1% agarose gel with DNA size markers.

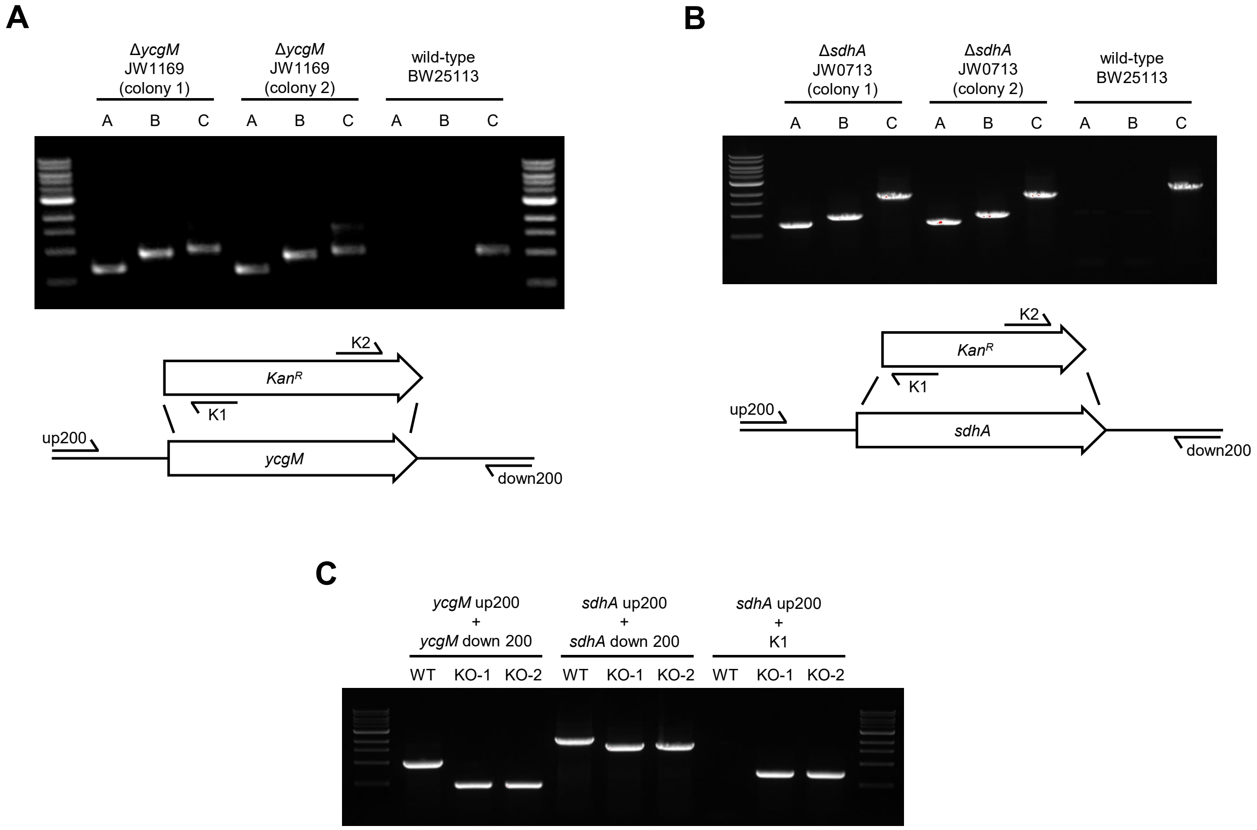

Fig. S12. Verification of the *E. coli* mutants used in this study. Genomic DNA was isolated from wild-type *E. coli* (BW25113), Keio collection strains JW1169 (Δ*ycgM*) and JW0713 (Δ*ycgM*), and Δ*ycgM*Δ*sdhA* double-mutant cells grown from single colonies. (**A** and **B**) PCR reactions contained the indicated DNA source and either primer set A (up200 and K1), B (down200 and K2) or C (up200 and down200). (**C**) PCR reactions contained the indicated primer set and DNA from either wild-type (WT) or Δ*ycgM*Δ*sdhA* double-mutants (KO-1 and KO-2). Reaction products were analyzed by electrophoresis on a 1% agarose gel with DNA size markers.

Supplemental Table 1. Oligonucleotide Primers Used in This Study

Expression Constructs		
Organism/Gene/Protein	Primer	Sequence (5'→3')
B. taurus FAHD2A	Bt FAHD2-tp Nde F	ggaattccatatggcacagaggtggccctttc
	Bt FAHD2 Xho R	ttccgctcgagtcacaccaccttgttgatgatgac
H. sapiens FAHD1	Hs FAHD1 Nde F	ggaattccatatgggaatcatggcagcatcc
	Hs FAHD1 Xho R	ttccgctcgagtcaatattctggcttttccactttaaatg
H. sapiens FAHD2A	Hs FAHD2-tp Nde F	ggaattccatatggctcagaagtggccctttcaacc
	Hs FAHD2 Xho R	ttccgctcgagtcacaccaccttgttgatgatgac
H. sapiens FAHD2B	Hs FAHD2B_CO Nde F	ggaattccatatgcgtttggtgcaattcc
	Hs FAHD2B_CO Xho R	ttccgctcgagttagaccactttgttgatgataacg
A. thaliana FAHD1 (At4g15940)	At4g15940-tp Nde F	ggaattccatatgcaagggacgaagatcgtctg
	At4g15940 Xho R	ttccgctcgagttagctcaagggctttacacg
A. thaliana FAHD2 (At3g16700)	At3g16700-tp Nde F	ggaattccatatgcaaggcacaaagatcgtcgg
	At3g16700 Xho R	ttccgctcgagctatctcaacaatggctttagccg
S. cerevisiae FMP41	Sc FMP41 Nhe F	ggaattcgctagcatgagctacaattatctgaaggc
	Sc FMP41 Eco R	cggaattctcacgtttctctaaattcatatgg
E. coli YcgM	Ec YcgM Nde F	ggaattccatatgtatcaacatcacaactggc
	Ec YcgM Xho R	ttccgctcgagttacaaaacgcgagttgtcaaag
B. subtilis YisK	Bs YisK Nhe F	ggaattcgctagcatgaaatttgcgacaggggaac
	Bs Yisk Xho R	ttccgctcgagttagccaatttggtttgacagcg
M. maripaludis FAHD2	Mm FAHD Nde F	ggaattccatatggaaataaacccttcaaaaatag
	Mm FAHD Xho R	ttccgctcgagtcattcaacgaaatttttcaaaattc
Complementation Constructs		
S. cerevisiae FMP41	Sc FMP41+pro Hind F	tacccaagcttcgagatagcgacggggttc
	Sc FMP41 Bam R	cgcggatccttatgtttctctaaattcatatggtcccg
E. coli YcgM	Ec YcgM Hind F	tacccaagcttatgtatcaacatcacaactggc
	Ec YcgM Xba R	gctctagattacaaaacgcgagttgtcaaag
E. coli SdhA	Ec SdhA Hind F	tacccaagcttatgaaattgccagtcagagaatttg
	Ec SdhA Xba R	gctctagattagtaagtacgaatcttcggc
M. maripaludis FAHD2	Mm FAHD Nsi F	aaaaatgcatggaaataaacccttcaaaaatag
	Mm FAHD Asc R	tcctaggcgcgccttattcaacgaaatttttcaaaattcc
Creating and Confirming Knockouts		
S. cerevisiae FMP41	YNL168C A	agtttatcaattgcttaagcctcct
	YNL168C B	agacttcttcgggtttcatcttagt
	YNL168C D	ctaacaactggctgaagagtcctta
	KanB	ctgcagcgaggagccgtaat
E. coli YcgM, SdhA	K1	ccagtcatagccgaatagcctc
	K2	cggtgccctgaatgaactgc
	Ec YcgM up200	caggcattaaccgagcaagg
	Ec YcgM down200	gatttcgtcggtgtcttcatcc
	Ec SdhA up200	tggtcggtttttcgctacc
	Ec SdhA down200	tgccgttcatgttcagaccg
M. maripaludis FAHD2	hpt9410-us-Fg	agatatccatcacactggcggccgcacaaagaaaaattaagcaacatc
	hpt9410-us-Rg	ttcaacgaaatttttcaaaattccaattaatttgctcatataattcacc
	hpt9410-ds-Fg	ggaattttgaaaaatttcgttgaataaattaatatttttatttttgtg
	hpt9410-ds-Rg	tatagggcgaattgggccctctagaagttccttcaacccagaaatttaatc
