# Supplementary material for: A universal metabolite repair enzyme removes a strong inhibitor of the TCA cycle
Source: Nat Commun. 2024 Jan 29;15:846. doi: 10.1038/s41467-024-45134-0 (PMC10825186; doi:10.1038/s41467-024-45134-0)
Supplement: Supplementary file 3 — Description of Additional Supplementary Files [file 41467_2024_45134_MOESM3_ESM.pdf]

Description of Additional Supplementary Files

File name: Supplementary Data 1

Description: Metabolomics summary

File name: Supplementary Data 2

Description: Metabolomics example calculation
