# Supplementary material for: A universal metabolite repair enzyme removes a strong inhibitor of the TCA cycle
Source: Nat Commun. 2024 Jan 29;15:846. doi: 10.1038/s41467-024-45134-0 (PMC10825186; doi:10.1038/s41467-024-45134-0)
Supplement: Supplementary file 6 — Reporting Summary [file 41467_2024_45134_MOESM6_ESM.pdf]

nature portfolio

Corresponding author(s): Thomas Niehaus

Last updated by author(s): Jan 4, 2024

Reporting Summary

Nature Portfolio wishes to improve the reproducibility of the work that we publish. This form provides structure for consistency and transparency in reporting. For further information on Nature Portfolio policies, see our Editorial Policies and the Editorial Policy Checklist.

Statistics

For all statistical analyses, confirm that the following items are present in the figure legend, table legend, main text, or Methods section.

n/a	Confirmed	
☐	☒	The exact sample size (*n*) for each experimental group/condition, given as a discrete number and unit of measurement
☐	☒	A statement on whether measurements were taken from distinct samples or whether the same sample was measured repeatedly
☐	☒	The statistical test(s) used AND whether they are one- or two-sided<br>*Only common tests should be described solely by name; describe more complex techniques in the Methods section.*
☐	☒	A description of all covariates tested
☐	☒	A description of any assumptions or corrections, such as tests of normality and adjustment for multiple comparisons
☐	☒	A full description of the statistical parameters including central tendency (e.g. means) or other basic estimates (e.g. regression coefficient) AND variation (e.g. standard deviation) or associated estimates of uncertainty (e.g. confidence intervals)
☐	☒	For null hypothesis testing, the test statistic (e.g. *F*, *t*, *r*) with confidence intervals, effect sizes, degrees of freedom and *P* value noted<br>*Give P values as exact values whenever suitable.*
☒	☐	For Bayesian analysis, information on the choice of priors and Markov chain Monte Carlo settings
☒	☐	For hierarchical and complex designs, identification of the appropriate level for tests and full reporting of outcomes
☒	☐	Estimates of effect sizes (e.g. Cohen's *d*, Pearson's *r*), indicating how they were calculated

Our web collection on statistics for biologists contains articles on many of the points above.

Software and code

Policy information about availability of computer code

Data collection	For metabolomics data, Xcalibur™ software version 2.1 (Thermo Scientific) was used for data collection. For spectrophotometric data, Cary UV Workstation software version 1.1.298 was used for data collection. For HPLC data, OpenLab ChemStation software version 2.19.20 was used for data collection.
Data analysis	Kinetic parameters were calculated by fitting data to the Michaelis–Menten equation using GraphPad Prism Software version 5.01. ImageJ software version 1.53t was used to measure the size of yeast and bacteria colonies using the 'analyze particles' function. Metabolomics data files were converted from .RAW to .mzML and .mgf formats using the ProteoWizard tool MSConvertGUI. MZmine version 2.53 was utilized for extracting exact-mass chromatographic data for isotope ratio calculations, for generating untargeted metabolite feature tables, and for tabulating peak heights from targeted exact masses for calculating relative signal intensity ratios.

For manuscripts utilizing custom algorithms or software that are central to the research but not yet described in published literature, software must be made available to editors and reviewers. We strongly encourage code deposition in a community repository (e.g. GitHub). See the Nature Portfolio guidelines for submitting code & software for further information.

Data

Policy information about availability of data

All manuscripts must include a data availability statement. This statement should provide the following information, where applicable:

- Accession codes, unique identifiers, or web links for publicly available datasets
- A description of any restrictions on data availability
- For clinical datasets or third party data, please ensure that the statement adheres to our policy

The metabolomics data generated in this study have been deposited to both the Metabolights database under accession code MTBLS8848 [www.ebi.ac.uk/metabolights/MTBLS8848] and the Data Repository for U of M under accession code 259320 [https://hdl.handle.net/11299/259320]. The biochemical and physiological data generated in this study are provided in the Supplementary Information/Source Data file.

Research involving human participants, their data, or biological material

Policy information about studies with human participants or human data. See also policy information about sex, gender (identity/presentation), and sexual orientation and race, ethnicity and racism.

Reporting on sex and gender	N/A
Reporting on race, ethnicity, or other socially relevant groupings	N/A
Population characteristics	N/A
Recruitment	N/A
Ethics oversight	N/A

Note that full information on the approval of the study protocol must also be provided in the manuscript.

Field-specific reporting

Please select the one below that is the best fit for your research. If you are not sure, read the appropriate sections before making your selection.

☒ Life sciences ☐ Behavioural & social sciences ☐ Ecological, evolutionary & environmental sciences

For a reference copy of the document with all sections, see nature.com/documents/nr-reporting-summary-flat.pdf

Life sciences study design

All studies must disclose on these points even when the disclosure is negative.

Sample size	For colony size determinations in yeast and bacterial strains, the sample size was 55-95. This sample size was used because it allowed for an ample number of colonies to be measured without influencing colony size due to overcrowding.
Data exclusions	No data was excluded from the analyses.
Replication	For metabolomics, 4 replicates were harvested for each treatment, which is a typical sample size for metabolomics analyses to ensure reproducibility. For enzyme assays, 3 replicates (from independent enzyme preparations) were measured to ensure accuracy and reproducibility. All attempts at replication were successful.
Randomization	Metabolomics samples were randomly analyzed to control for analytical drift. For enzyme assays, randomization was not feasible as enzymes had to be freshly prepared individually and assayed immediately.
Blinding	Experimental blinding was done when preparing and analyzing metabolomics samples. For enzyme assays, experimental blinding was not feasible since enzymes and reagents had to be freshly prepared and then assays performed immediately by a skilled researcher.

Reporting for specific materials, systems and methods

We require information from authors about some types of materials, experimental systems and methods used in many studies. Here, indicate whether each material, system or method listed is relevant to your study. If you are not sure if a list item applies to your research, read the appropriate section before selecting a response.

Materials & experimental systems

n/a	Involved in the study
☒	☐ Antibodies
☐	☒ Eukaryotic cell lines
☒	☐ Palaeontology and archaeology
☒	☐ Animals and other organisms
☒	☐ Clinical data
☒	☐ Dual use research of concern
☐	☒ Plants

Methods

n/a	Involved in the study
☒	☐ ChIP-seq
☒	☐ Flow cytometry
☒	☐ MRI-based neuroimaging

Eukaryotic cell lines

Policy information about cell lines and Sex and Gender in Research

Cell line source(s)	Yeast cell lines were provided by Horizon Discovery. No vertebrate cell lines were used in this study.
Authentication	All yeast and bacterial cell lines were PCR verified before use to ensure expected genotypes.
Mycoplasma contamination	Cell lines were not tested for mycoplasma contamination.
Commonly misidentified lines (See ICLAC register)	N/A

Dual use research of concern

Policy information about dual use research of concern

Hazards

Could the accidental, deliberate or reckless misuse of agents or technologies generated in the work, or the application of information presented in the manuscript, pose a threat to:

No	Yes
☒	☐ Public health
☒	☐ National security
☒	☐ Crops and/or livestock
☒	☐ Ecosystems
☒	☐ Any other significant area

Experiments of concern

Does the work involve any of these experiments of concern:

No	Yes
☒	☐ Demonstrate how to render a vaccine ineffective
☒	☐ Confer resistance to therapeutically useful antibiotics or antiviral agents
☒	☐ Enhance the virulence of a pathogen or render a nonpathogen virulent
☒	☐ Increase transmissibility of a pathogen
☒	☐ Alter the host range of a pathogen
☒	☐ Enable evasion of diagnostic/detection modalities
☒	☐ Enable the weaponization of a biological agent or toxin
☒	☐ Any other potentially harmful combination of experiments and agents

Plants

Seed stocks	Arabidopsis genes were cloned from cDNA derived from Columbia-0 (WT) plants obtained from the Arabidopsis Biological Resource Center (ABRC), catalog number: CS60000
Novel plant genotypes	Plant genotypes were not measured in this study.
Authentication	Genes cloned from Arabidopsis were verified by Sanger sequencing.
